# Temperature is a poor proxy for synergistic climate forcing of plankton evolution

**DOI:** 10.1098/rspb.2018.0665

**Published:** 2018-07-18

**Authors:** Anieke Brombacher, Paul A. Wilson, Ian Bailey, Thomas H. G. Ezard

**Affiliations:** 1 Ocean and Earth Science, National Oceanography Centre Southampton, University of Southampton, Waterfront Campus, European Way, Southampton SO14 3ZH, UK; 2 Camborne School of Mines and Environmental Sustainability Institute, University of Exeter, Penryn Campus, Cornwall TR10 9FE, UK; 3 Biological Sciences, University of Southampton, Life Sciences Building 85, Highfield Campus, Southampton SO17 1BJ, UK

**Keywords:** microevolution, temperature, CO_2_, productivity, foraminifera, abundance

## Abstract

Changes in biodiversity at all levels from molecules to ecosystems are often linked to climate change, which is widely represented univariately by temperature. A global environmental driving mechanism of biodiversity dynamics is thus implied by the strong correlation between temperature proxies and diversity patterns in a wide variety of fauna and flora. Yet climate consists of many interacting variables. Species probably respond to the entire climate system as opposed to its individual facets. Here, we examine ecological and morphological traits of 12 633 individuals of two species of planktonic foraminifera with similar ecologies but contrasting evolutionary outcomes. Our results show that morphological and ecological changes are correlated to the interactions between multiple environmental factors. Models including interactions between climate variables explain at least twice as much variation in size, shape and abundance changes as models assuming that climate parameters operate independently. No dominant climatic driver can be identified: temperature alone explains remarkably little variation through our highly resolved temporal sequences, implying that a multivariate approach is required to understand evolutionary response to abiotic forcing. Our results caution against the use of a ‘silver bullet’ environmental parameter to represent global climate while studying evolutionary responses to abiotic change, and show that more comprehensive reconstruction of palaeobiological dynamics requires multiple biotic and abiotic dimensions.

## 1. Introduction

Changes in biodiversity are often linked to climate change, usually temperature. Phanerozoic species richness covaries with global temperature [1,2]. Cenozoic diversity patterns of mammals [3,4], plants [5,6], insects [6], plankton [7,8] and benthic microfauna [9,10] correlate with the high-latitude climate signal recorded in the *δ*^18^O composition of benthic foraminifera [11]. These results imply a dominant mechanism shaping biodiversity dynamics through time. Yet climate consists of many interacting variables, and species probably respond to the entire climate system as opposed to separate variables: Harnik *et al*. [12] argued that simultaneous changes in multiple environmental parameters drove most Phanerozoic extinction events, while Garcia *et al*. [13] show that increased threats on modern biodiversity become apparent when incorporating multiple dimensions of climate change. However, the extent to which the impact of abiotic forcing on within-species evolutionary change is underestimated when only single environmental factors are assessed remains largely unknown. Evidence exists for both synergistic (combined effects of multiple drivers are greater than the sum of individual drivers) and antagonistic (combined effects of multiple drivers are smaller than the sum of individual drivers) processes in modern ecosystems [14–16], but no empirical data exist for microevolutionary processes in deep time.

To accurately quantify the link between long-term (greater than 10 000 years) microevolution and climate change, high-resolution fossil records of multivariate evolutionary change need to be allied to multivariate reconstructions of local environmental conditions. Such data are rarely available. One of the few media on which multivariate evolutionary and environmental change can be determined at high temporal resolution is the marine fossil record of planktonic foraminifera. The excellent preservation of this group in open ocean sediments permits direct comparison of morphological and ecological change to high-resolution records of climate and evolution reconstructed from the same marine cores. Several studies have shown responses of foraminiferal morphology to sea surface temperature [17–20], but many have also reported relationships with productivity [21] and ocean stratification [17,22]. However, none of these studies analysed the ecological and evolutionary impacts due to the interplay of multiple climate drivers.

**Figure 1.**
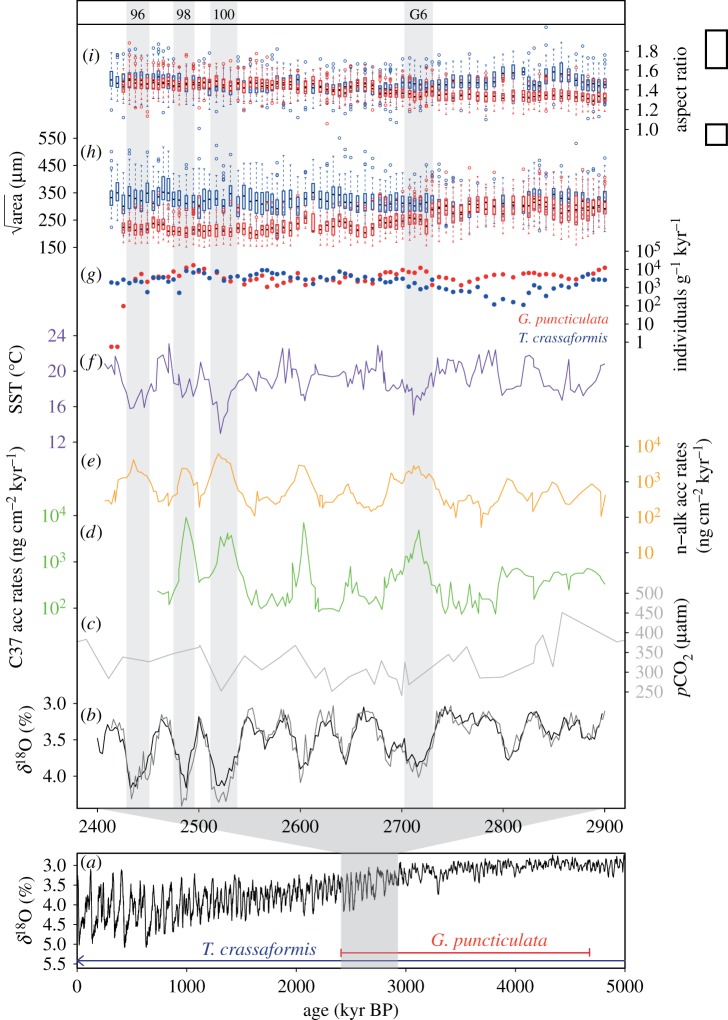
Environmental reconstructions and morphology of two planktonic foraminifera species at IODP Site U1313: oxygen isotopes from the Lisiecki *et al*. [26] benthic stack (*a*,*b*, black lines) and Site U1313 [27] (*b*, grey line), atmospheric CO_2_ reconstructed at ODP Site 999 by Martínez-Botí *et al*. [24] (*c*), productivity (*d*), aeolian input (*e*) and sea surface temperature (*f*) by Naafs *et al*. [28], abundance (*c*) of *Globoconella puncticulata* (red) and *Truncorotalia crassaformis* (blue) (this study), and size (*d*) and shape (*e*) of *G. puncticulata* and *T. crassaformis* [29]. Key glacial stages are indicated by grey bars.

Here, we study species' responses to multivariate climate change during the last great climate transition in Earth's history: the late Pliocene to earliest Pleistocene intensification of Northern Hemisphere glaciation (3.6–2.4 Ma) [23]. This interval was characterized by major reorganizations of the global climate system: global atmospheric CO_2_ concentrations [24] dropped below the approximately 280 µatm threshold for extensive Northern Hemisphere glaciation [25] between 2.9 and 2.7 Ma (figure 1*c*). By 2.7 Ma, continental ice-sheets had expanded significantly on Greenland, Scandinavia and North America as evidenced by the onset of widespread ice-rafted debris deposition in high northern latitude oceans [30,31] and an increase in the amplitude of glacial–interglacial cycles as recorded in benthic foraminifera oxygen isotopes (*δ*^18^O to greater than 0.5‰) from Marine Isotope Stage (MIS) G6 (2.7 Ma) onwards (figure 1*a,b*). In the North Atlantic Ocean this transition to deeper glacials was associated with (i) incursions of southern-sourced deep waters [32], (ii) a major intensification of dust flux from North America carried on the westerly winds [28,33], and (iii) increases in glacial primary productivity [28,34] (figure 1*d*,*e*). Together, these synergistic environmental changes probably had a major impact on life in the marine realm [35]. All environmental parameters would have directly influenced individual foraminifera during their lifetime: species prefer specific temperature ranges [36,37] and will respond to temperature changes in their environment [19,20] as well as productivity regimes [38], while ocean pH influences calcification potential [38]. To quantify the combined effects of changes in temperature, primary productivity, dust input and atmospheric CO_2_ on evolution during the intensification of Northern Hemisphere glaciation, we employ multivariate statistical techniques to compare ecological (abundance, figure 1*g*) and morphological (size and shape, figure 1*h*,*i*) dynamics across 12 629 specimens of the ecologically similar planktonic foraminifera species *Globoconella puncticulata* and *Truncorotalia crassaformis* (electronic supplementary material, figure S1). *Truncorotalia crassaformis* survived the intensification of Northern Hemisphere glaciation and is still alive today, whereas *G. puncticulata* became extinct shortly after 2.41 Ma (during MIS 96 [39]). These two foraminifera species provide an opportunity to study species' responses to multivariate climate change under contrasting evolutionary outcomes.

## 2. Material and methods

### (a) Study species

*Truncorotalia crassaformis* and *G. puncticulata* (electronic supplementary material, figure S1) are two ecologically similar species characterized by low trochospiral shells with flattened spiral sides, inflated umbilical sides and umbilical–extraumbilical apertures [40]. Both inhabit thermocline to subthermocline waters at middle and low latitudes [40,41]. *Truncorotalia crassaformis* originated around 5.7 Ma and survives to the present day. *Globoconella puncticulata* first appeared around 4.6 Ma and became extinct at 2.41 Ma [39], shortly after the onset of significant Northern Hemisphere glaciation at 2.72 Ma [31]. Our 500 000-year study interval includes the onset of widespread Northern Hemisphere glaciation (MIS G6, 2.72 Ma, [31]), the first three major Northern Hemisphere glaciations MIS 100, 98 and 96 [26], and ends with the extinction of *G. puncticulata* [39]. Preservation of planktonic foraminifera is good throughout the study interval [42] implying little dissolution effects on traits. We study three traits: mean shell area and mean aspect ratio per time slice (data from [29]), which have been shown to be repeatable proxies for shell size and shape [43], and abundance (this study) (figure 1*g–i*). Schmidt *et al*. [44] show that maximum size and abundance generally occur at the same temperature for modern planktonic foraminifera species, implying that the combination of abundance and size are indicators of ecological optima [44,45]. Shell shape controls the area : volume ratio which influences respiratory processes according to first principles of cell physiology.

### (b) Study site

IODP Site U1313 is located in the mid latitude North Atlantic Ocean at the base of the upper western flank of the Mid-Atlantic Ridge at a water depth of 3426 m (41° N, 32.5′ W) on the northern edge of the North Atlantic subtropical gyre (electronic supplementary material, figure S2). The sediments deposited at Site U1313 accumulated at consistently high rates (approx. 5 cm kyr^−1^) for the past 5 Myr [26,42], and yield a demonstrably continuous record of sedimentation through the intensification of Northern Hemisphere glaciation [27] and exceptionally well-preserved microfossil carbonate [33].

We used 75 sediment samples from Site U1313 (every 30 cm, i.e. approximately 5-kyr-resolution) dated by Bolton *et al*. [27] by matching an orbital-resolution benthic foraminiferal *δ*^18^O record to the global oxygen isotope stack [26]. The samples were dry-sieved over a greater than 150 µm mesh sieve and divided into equal fractions using a microsplitter until a single fraction contained 70–150 specimens of *T. crassaformis* or *G. puncticulata*. The smallest analysed individual of *T. crassaformis* is 30% larger than the smallest particle that could be captured by the sieve, so it is unlikely we missed any specimens of this species by our choice of size fraction. For *G. puncticulata* the smallest possible particle to be captured by the sieve is smaller than the species’ mean shell size minus 2 sigma, meaning greater than 97.5% of all specimens would be captured by the current size fraction, implying that the used size fraction has little effect on the data. To avoid size bias, all individuals from a single fraction were analysed, resulting in a total of 12 633 individuals (6058 specimens of *T. crassaformis* and 6575 of *G. puncticulata*) over the studied interval. The total number of specimens in each sample was estimated by multiplying the number of individuals found in the fraction by the total number of fractions into which the sample was split. Abundance (represented as accumulation rates) was calculated as the number of individuals divided by the weight of the sediment size fraction greater than 150 µm^2^, divided by the total time in the sample as determined by Bolton *et al*. [27]. Morphological trait data are available in the Dryad database as part of [29]. Abundance data are deposited in the Figshare repository at https://figshare.com/s/9db6657150242fb8a593 and will be made publicly available upon manuscript acceptance.

### (c) Existing environmental reconstructions

When comparing biotic to abiotic processes, global climate is often represented by oxygen isotope records generated from foraminiferal calcite. However, these records form a composite of seawater temperature, salinity and global ice volume, and mainly represent high-latitude climate. Therefore, to directly compare species' responses to their immediate environment, local climatic reconstructions are required. Several published orbitally resolved environmental reconstructions are available for Site U1313, including n-alkane accumulation rates representing mixed-layer productivity [46], terrestrial plant leaf wax fluxes linked to aeolian input of North American dust [28] and a mean annual sea surface temperature record based on the saturation index of C_37_ alkenones (U^k’^_37_) [28]. Although our study species inhabit thermocline waters, a comparison of foraminifera test Mg/Ca ratio-derived sea surface and thermocline temperatures over the interval approximately 2.4–2.6 Ma (CT Bolton 2018, personal communication) showed similar morphological response between our study species, which agrees with findings from a study by Schmidt *et al*. [47] showing similar response to temperature in species living at different depth habitats. Two plant wax records are available for Site U1313, one based on n-alkanes and the other on C26-alkan-1-ol chains. The two records are highly correlated [28] and argued to be from a common North American origin [28]. As both are therefore likely to experience the same absolute level of noise, we chose to use the n-alkanes record because its values are higher by a factor approximately 1.5 when compared with the C26-alkan-1-ol-based record, providing the highest signal : noise ratio. At present, the North Atlantic Subtropical Gyre is nutrient-limited with nitrogen fixation correlated to dissolved iron [48] and the strong correlation between aeolian input and productivity in the late Pliocene (figure 1*d*,*e*) implies that this was to an extent also true for our study interval. Biotic responses were compared to the site-specific reconstructions of sea surface temperature, productivity and dust input [28,46], and a global reconstruction of atmospheric CO_2_ concentration [24] to represent multiple dimensions of environmental conditions experienced by the study species (figure 1*c*–*f*). Although reconstructed from an equatorial site, the atmospheric CO_2_ reconstruction is also likely to reflect changes in pH at IODP Site U1313 induced by atmospheric CO_2_ given the short mixing time of CO_2_ between the sea surface and the atmosphere [24]. Additionally, Site U1313 probably experienced little oceanographic change during the intensification of Northern Hemisphere glaciation [49], implying a constant local CO_2_ balance. Aeolian dust is used here to indicate nutrient levels, as dust provides an additional nutrient source to the oligotrophic and iron-limited subtropical gyre [48], and ocean pH influences calcification potential, influencing selection for larger shell size and thickness with decreasing pH [38]. Although these parameters only represent a subset of all environmental change, comparing species' responses to these parameters and their combinations will shed new light on multivariate drivers of evolutionary change.

### (d) Analysis

**Figure 2.**
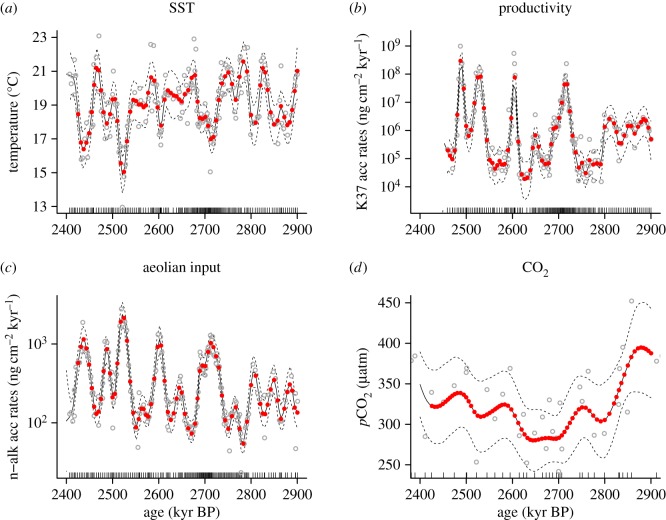
Generalized additive models (GAMs) used to interpolate values of sea surface temperature (*a*), productivity (*b*), aeolian dust input (*c*) and atmospheric CO_2_ concentration (*d*) at the ages of the foraminifera samples from Site U1313 (internal tick marks on *x*-axis). Original data points are denoted by open circles, with solid and dashed lines representing the GAM and 95% confidence interval, respectively. Estimated values are indicated by red circles.

Because the environmental reconstructions of Site U1313 and the foraminifera trait data were generated using different sample sets, the climate data point ages are offset relative to our foraminifera samples. Generalized additive models (GAMs) were employed to interpolate the climate parameters to the foraminiferal sample ages. The individual climate records were smoothed using a GAM, and the value at the age of the foraminifera samples was estimated using the non-parametric curve (figure 2). To enable comparisons of responses among traits, we studied the morphological trait means and single abundance values per time slice. To compare trait changes to climate change, first differences of all biotic and environmental records were calculated to remove temporal autocorrelation in the residuals (electronic supplementary material, figures S3 and S4). Using linear models, the first difference of the trait records were then compared to those of the environmental parameters to calculate the total variance explained in the biotic parameters to change in the environmental parameters and their interactions. Trait variance explained by individual parameters was calculated as the variance explained (*R*^2^) by the full model (up to and including all two-way interactions), minus the variance explained by the model with each parameter removed [50]. Another linear model with only univariate effects was compared to our full model to quantify the synergistic effects of interactions among climate variables on morphological and ecological change. We focus on the *R*^2^ value due to its tractability, and the possibility to study effect sizes of all climate variables and their interactions. *Δ*Akaike information criterion (AIC) scores of individual parameters and interactions are included in electronic supplementary material, figure S5.

## 3. Results

**Figure 3.**
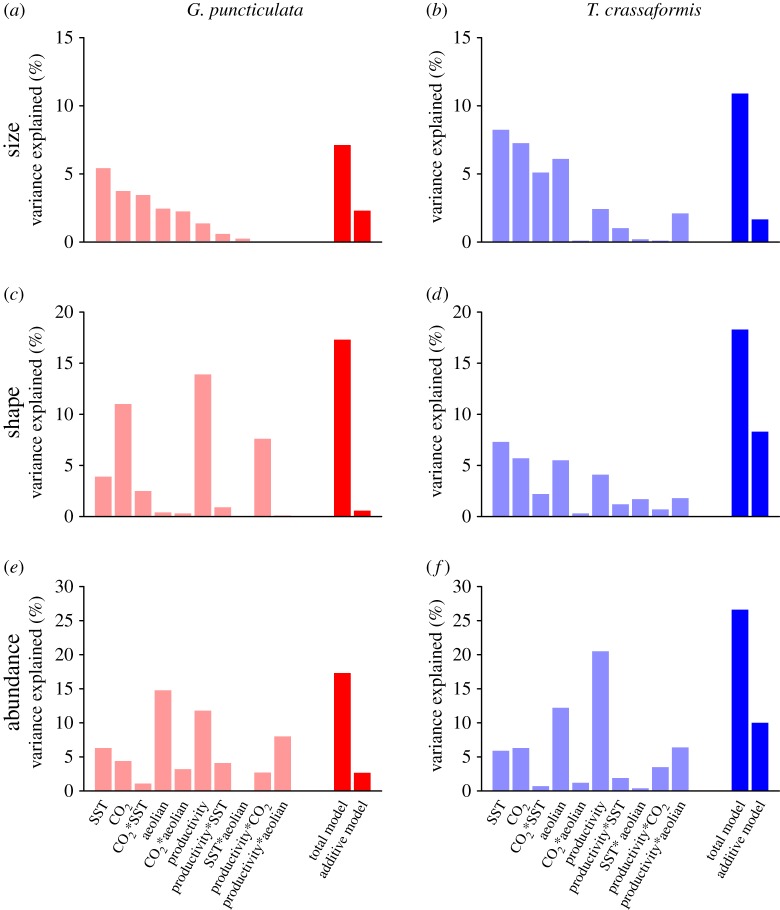
Variance explained in size (*a*,*b*), shape (*c*,*d*) and abundance (*e*,*f*) of *Globoconella puncticulata* (red) and *Truncorotalia crassaformis* (blue) from North Atlantic Site U1313 (41̊N) by the environmental parameters and their interactions. (Online version in colour.)

In all cases, most variation of that explained by models was through the combination of all studied parameters and their interactions (7.1%, 17.3% and 17.3% for *G. puncticulata* size, shape and abundance, and 10.9%, 18.3% and 26.6% for *T. crassaformis* size, shape and abundance). No single driver is found to dominate the variance explained in all studied traits (figure 3). Variation in size of *G. puncticulata* and size and shape of *T. crassaformis* are most strongly correlated to temperature (5.5%, 8.2% and 7.3% for *G. puncticulata* size, and *T. crassaformis* size and shape, respectively), whereas productivity is most strongly correlated to shape in *G. puncticulata* (13.9% variance explained) and abundance of *T. crassaformis* (20.5% variance explained). Abundance of *G. puncticulata* is best explained by aeolian input (14.8% variance explained). However, in all three cases little variance is explained by these parameters alone.

The model including all two-way interactions provides a significantly better fit to the data than the additive model without the interactions for shape in *G. puncticulata* (ANOVA, *F*_6,69_ > 2.1, *p* < 0.05), and abundance in *T. crassaformis* (ANOVA, *F*_6,69_ > 2.4, *p* < 0.05). In both species, response of abundance is most strongly correlated to the environmental parameters (Wilcoxon signed-rank test, *p* < 0.01 and *p* < 0.05 for *G. puncticulata* and *T. crassaformis,* respectively), but no difference was detected between the responses of size and shape (Wilcoxon signed-rank test, *p* = 0.79 and *p* = 0.74 for *G. puncticulata* and *T. crassaformis*, respectively). Response of size is stronger in *G. puncticulata* than *T. crassaformis* (Wilcoxon signed-rank test, *p* < 0.01), but the strength of responses is comparable between species for shape and abundance (Wilcoxon signed-rank test, *p* = 0.65 for shape, *p* = 0.69 for abundance).

## 4. Discussion

Our results show that temperature is a poor proxy for synergistic climate forcing of the observed biotic change. The amount of morphological and ecological variation explained is highest when studied including interactions between multiple environmental parameters. These results imply that species’ response to climate change can be underestimated when only single variables are taken to represent the complex multifaceted climate system: in our study the amount of biotic variance explained by environmental change decreases by up to a factor approximately 2 if only single variables are considered (figure 3), and is likely to decrease further relative to multivariate change with more drivers included in the analyses. Our findings are consistent with short-term studies of modern populations that show increased mortality as a response to multiple environmental stressors [14,15,51], as well as macroevolutionary research into the abiotic drivers of mass extinctions [12,13]. The strength of the correlation between environmental parameters and traits varies—no single parameter best explains the variance in all records. Therefore, our results caution against the use of a single ‘silver bullet’ environmental parameter to represent global climate while studying evolutionary response to abiotic change.

Our results generate an appropriately multi-faceted picture of abiotic forcing, and suggest strongly that (sea surface) temperature alone is a poor proxy for environmental changes that supposedly drive ecological and morphological changes through time. These results contrast with the findings of spatial studies by Tittensor *et al*. [52], Rutherford *et al*. [53] and Fenton *et al*. [54], who used multiple species of planktonic foraminifera to report the dominance of temperature in shaping ecological processes across space. The comparison of these results implies that spatial abiotic drivers [54] do not directly translate to those operating through time along single species' branches, supporting hypotheses that spatial variation is not a suitable substitute for temporal change and that data with a substantial temporal component are required to accurately reconstruct biodiversity dynamics over long timescales [55,56].

Neither species’ responses are synergistic (total response > sum of response to individual parameters) because response to the total model describes less trait variance than the sum of the responses to single climate variables. These results are consistent with the findings of Darling *et al*. [16], who reviewed 112 published mortality experiments and found only a third showed synergistic responses to external drivers. In our case, the species' antagonistic responses (total response < sum of response to individual parameters) to abiotic change could be explained by a common driving mechanism underpinning the studied environmental variables. Late Pliocene North Atlantic sea surface temperature, productivity, aeolian dust input and CO_2_ are all correlated and strongly linked to the intensification of Northern Hemisphere glaciation [24,28,31–33,46], resulting in similar trends in each record (figure 1*c*–*f*) that are expected to add little extra variance explained in the biotic records. Depending on its ecological preferences, a species could respond to parameters in opposite ways: a positive response to an increase in one variable and a negative response to increase in another could lead to little net effect when both variables increase, decreasing the variance explained by the total model. This further advocates the use of multiple environmental parameters in the model as it allows exploration of synergistic or antagonistic responses that would otherwise have remained unknown.

The unexplained variance in size, shape and abundance dynamics could be attributable to several factors. Firstly, planktonic foraminifera have a lifespan of a few weeks [38]. Individuals living in different seasons in the mid-latitude Atlantic Ocean experience temperature differences of up to 6–7°C [57]. Such variability is comparable to mean annual Late Pliocene–Early Pleistocene glacial–interglacial SST changes at our study site [46,49] (figure 1*f*), and plastic responses to these seasonal differences could increase trait variance in our time-averaged samples. Secondly, some of the observed trait variance could be caused by migration of morphologically distinct populations. However, the position of major surface water currents probably remained unchanged throughout our study interval [49], providing little opportunity for migrations of populations from other areas. Third, abundance and shell shape responded more strongly to the studied environmental variables than shell size, but in reality traits are often not independent [58,59]. Such covariation can constrain evolutionary responses to environmental drivers [60]. Climatic upheaval can disrupt the covariation between traits [29], emphasizing the need for comprehensive understanding of abiotic catalysts for biotic change.

## 5. Conclusion

We show that morphological and ecological change through time correlate to multivariate environmental change, particularly the interactions between distinct parts of global climate. No single climate variable was identified that best explained morphological and ecological change in all studied traits of both foraminifera species, implying that responses to environmental change are likely to be severely underestimated when only single variables such as temperature are used to represent global climate. Temperature was not even the most important single climate variable explaining morphological or ecological variation. Responses also varied among morphological and ecological traits, suggesting trait-specific sensitivities to environmental change that require comprehensive comparative analyses to tease apart. Our results imply that use of local temperature as a single variable to test for biotic response to climate change is limiting. Successful reconstruction of eco-evolutionary dynamics in deep time therefore necessitates multivariate explanatory and response variables.

## Supplementary Material

Supplementary figures S1-S5

## Data accessibility

Abundance data are available from Figshare https://figshare.com/s/9db6657150242fb8a593.
