# Supplementary material for: Temperature is a poor proxy for synergistic climate forcing of plankton evolution
Source: Proc Biol Sci. 2018 Jul 18;285(1883):20180665. doi: 10.1098/rspb.2018.0665 (PMC6083249; doi:10.1098/rspb.2018.0665)
Supplement: Supplementary figures S1-S5 [file rspb20180665supp1.pdf]

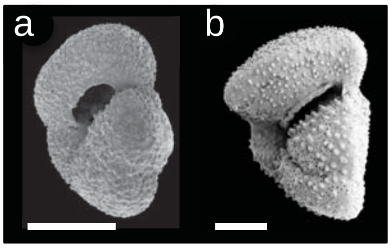

Figure S1
Study species *Globoconella puncticulata* (a) and *Truncorotalia crassaformis* (b). The scale bars represent 100 µm.

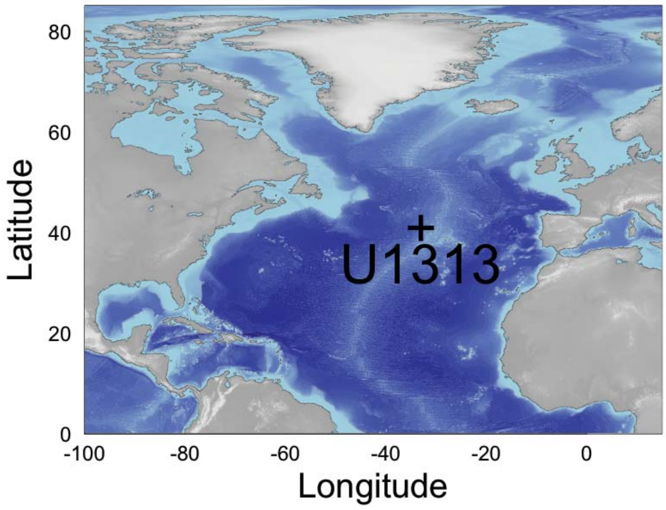

Figure S2
Map of study site IODP Site U1313 in the North Atlantic

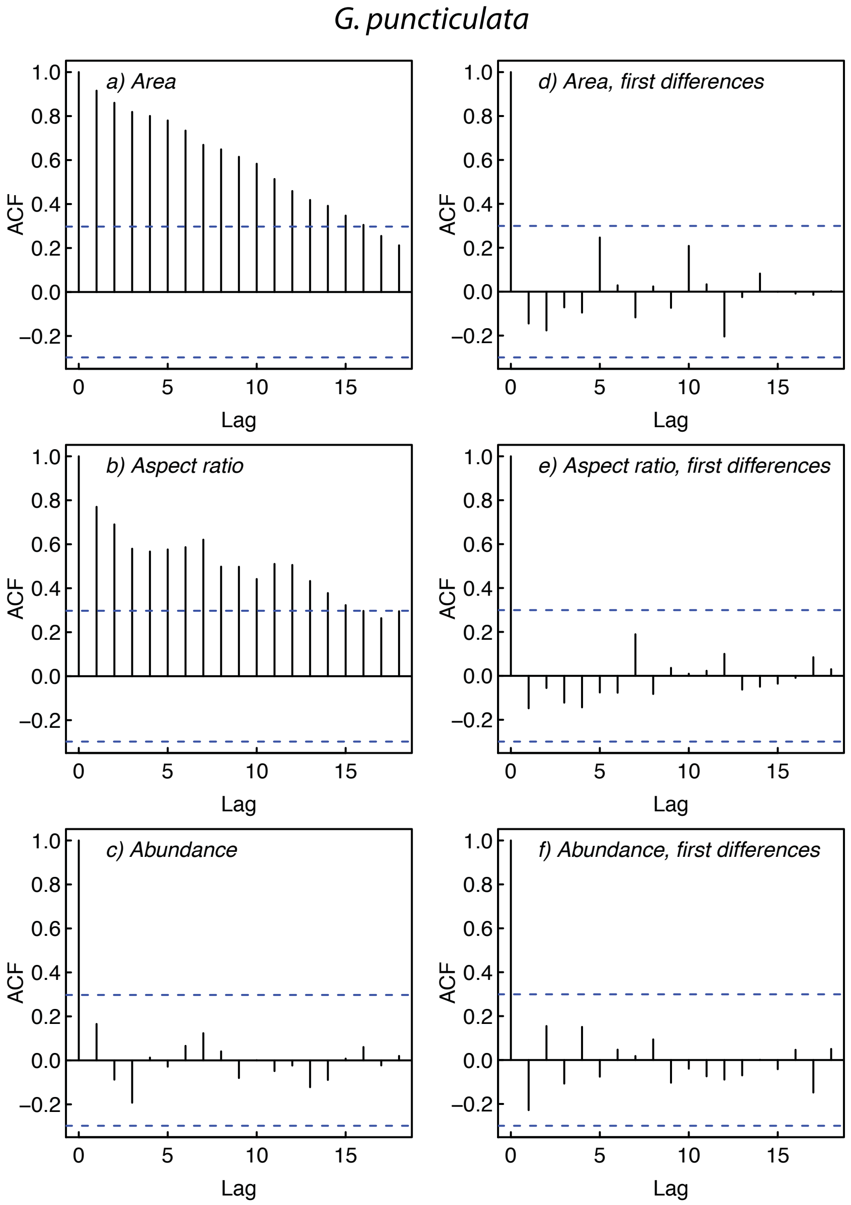

Figure S3
Autocorrelation plots for traits of *Globoconella puncticulata* showing autocorrelation in the original time series (a-c) but not in the first differences (d-f)

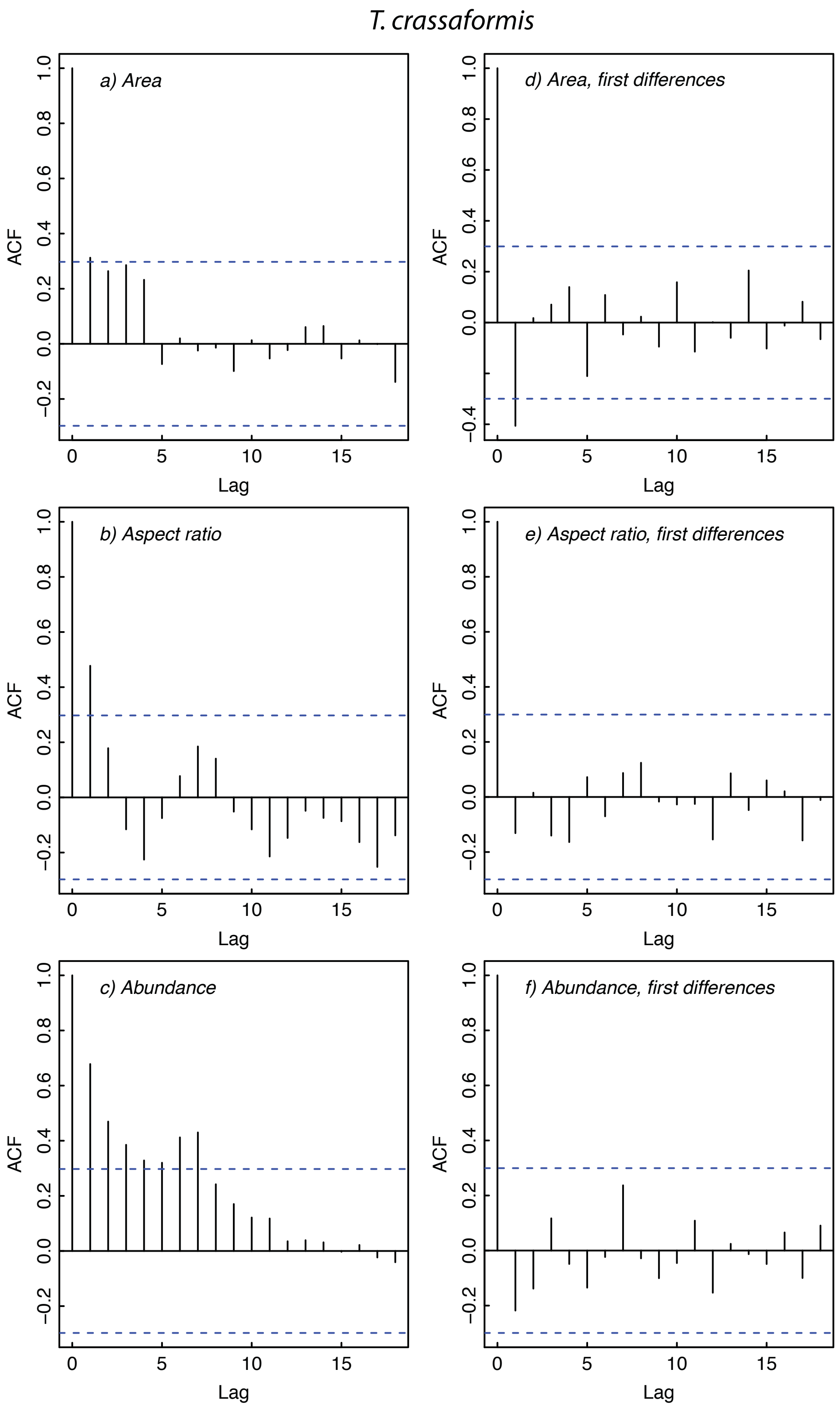

Figure S4
Autocorrelation plots for traits of *Truncorotalia crassaformis* showing autocorrelation in the original time series (a-c) but not in the first differences (d-f). Only area in T. crassaformis still contained a small amount of autocorrelation in the first differences time series. However, comparison of two Generalized Least Squares models with and without autocorrelation (by setting the continuous auto-correlation parameter phi=0.3 and phi=0.1, which converges to 0 under maximum likelihood, respectively) show lower AIC values for the model without autocorrelation, implying that the model with temporal autocorrelation is actually a poorer fit to the data than that without.

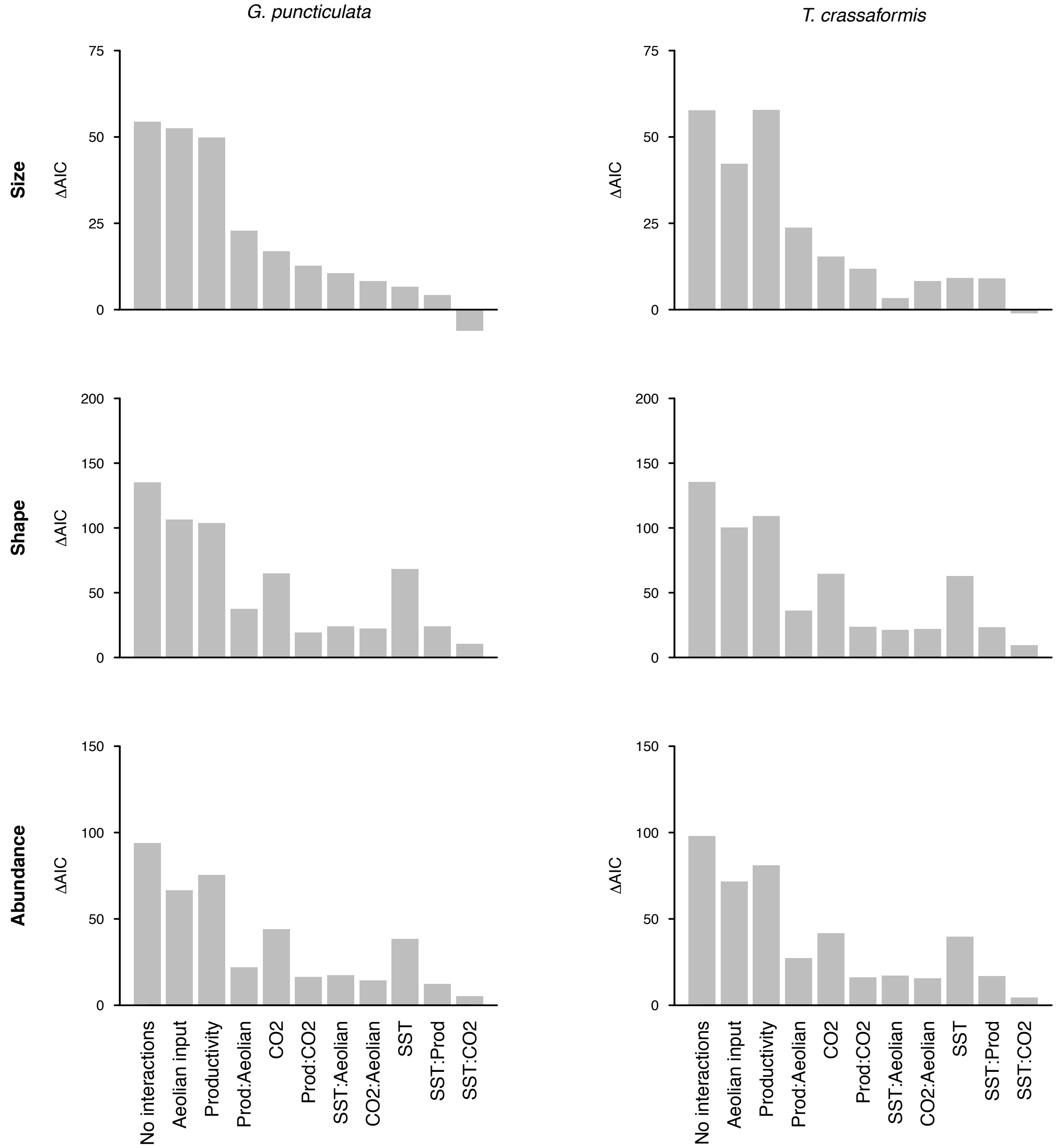

Figure S5

ΔAIC values showing the added relative importance of each environmental parameter or combination of parameters to the total model
